# Safer ICU trainee handover: a service improvement project

**DOI:** 10.1186/cc11125

**Published:** 2012-03-20

**Authors:** E Godfrey, I Hassan, A Carson-Stevens, AG Saayman

**Affiliations:** 1 University Hospital of Wales, Cardiff, UK; 2 Cardiff University, Cardiff, UK

## Introduction

Quality handover between team members within the ICU is vital for patient safety. Critically ill patients are at high risk of medical errors; these complex patients are exposed to high-risk interventions, medical and procedural [1]. Distractions are known to be particularly prevalent within critical care [2]. This can compromise handover efficiency, interrupt information-giving and may ultimately lead to poorer patient outcomes [3]. We sought to demonstrate the capability of junior physicians to lead change to their practices that benefit the quality of patient care in a large critical care unit. We present an improvement project that has transformed handover quality in our ICU.

## Methods

Participant observation of handover practices took place within a high-occupancy 33-bed adult ICU. Quantitative assessment of handover criteria as per Royal College of Anaesthetists guidelines [4] was performed at baseline (handovers: *n *= 6, patients: *n *= 119) and 3 months post-intervention (handovers: *n *= 4, patients: *n *= 108). Interventions included presentation of data at multiprofessional departmental meetings, education of team members regarding frequency of handover interruptions and development and utilisation of an electronic handover tool.

## Results

Provision of patient details during handover was substandard. Utilisation of a structured handover sheet significantly improved the number of patient details provided; in particular, patient age (18% vs. 100%), duration of stay (29% vs. 79%) and medical management plan (53% vs. 93%). Frequent handover interruptions seen on initial observation significantly improved (100% vs. 25% of handover periods interrupted) following our collaboration with the senior nurse, physiotherapist and other team leads regarding the number and nonurgent nature of interruptions; at re-audit, interruptions occurred for clinically urgent requests only.

## Conclusion

Simple measures instituted by junior doctors, such as team education and use of a structured handover tool, can aid high-quality handover within critical care. Evidence suggests that high-quality handover within critical care will translate into improved clinical care for patients.
